# Jasmonic acid (JA) and gibberellic acid (GA_3_) mitigated Cd-toxicity in chickpea plants through restricted cd uptake and oxidative stress management

**DOI:** 10.1038/s41598-021-98753-8

**Published:** 2021-10-05

**Authors:** Parvaiz Ahmad, Vaseem Raja, Muhammed Ashraf, Leonard Wijaya, Andrzej Bajguz, Mohammed Nasser Alyemeni

**Affiliations:** 1 Botany and Microbiology Department, College of Science, King Saud University, Riyadh, 11451 Saudi Arabia; 2 Department of Botany, S.P. College, Srinagar, Jammu and Kashmir India; 3 Government Degree College for Women, Pulwama, Jammu and Kashmir 192301 India; 4 University of Agriculture, Faisalabad, Faisalabad, Pakistan; 5 Department of Biology and Ecology of Plants, Faculty of Biology, University of Bialystok, 15-245 Bialystok, Poland

**Keywords:** Gibberellins, Jasmonic acid

## Abstract

Cadmium stress is one of the chief environmental cues that can substantially reduce plant growth. In the present research, we studied the effect of jasmonic acid (JA) and gibberellic acid (GA_3_) applied individually and/or in combination to chickpea (*Cicer arietinum*) plants exposed to 150 µM cadmium sulphate. Cadmium stress resulted in reduced plant growth and pigment contents. Moreover, chickpea plants under cadmium contamination displayed higher levels of electrolytic leakage, H_2_O_2,_ and malonaldehyde, as well as lower relative water content. Plants primed with JA (1 nM) and those foliar-fed with GA_3_ (10^–6^ M) showed improved metal tolerance by reducing the accumulation of reactive oxygen species, malonaldehyde and electrolytic leakage, and increasing relative water content. . Osmoprotectants like proline and glycinebetaine increased under cadmium contamination. Additionally, the enzymatic activities and non-enzymatic antioxidant levels increased markedly under Cd stress, but application of JA as well as of GA_3_ further improved these attributes. Enzymes pertaining to the ascorbate glutathione and glyoxylase systems increased significantly when the chickpea plants were exposed to Cd. However, JA and GA_3_ applied singly or in combination showed improved enzymatic activities as well as nutrient uptake, whereas they reduced the metal accumulation in chickpea plants. Taken together, our findings demonstrated that JA and GA_3_ are suitable agents for regulating Cd stress resistance in chickpea plants.

## Introduction

Most often plants in natural environments are exposed to numerous ecological stresses that impede growth and productivity. Among those stresses, heavy metal toxicity is considered to be a prime factor for soil and water pollution^1,2^. Modern agricultural practices and rapid industrialization are the major contributors of heavy metals to the soil^3,4^. Being a non-essential heavy metal, cadmium (Cd) has the highest solubility and availability, therefore, it is absorbed by the plants with very much ease^5,6^. Cadmium invades into our surroundings through several natural and anthropogenic processes like weathering of rocks, subsurface mining, thermal power generation, and disproportionately employed wastewater and sewage water for irrigation practices in agriculture^3,7^. In plants, primary visible symptoms of Cd toxicity include necrosis, chlorosis, stunted growth of roots and shoots, and ultrastructural damage^6,8^. Cadmium can inhibit several significant plant activities such as membrane integrity, physiological aspects, metabolic and biochemical reactions, like photosynthesis and respiratory electron transport chain in the mitochondrion and chloroplast. Besides, it severely affects mineral nutrition and water balance^9,10^. With adaptive and resistance behavior, plants have well developed several endogenous stress tolerance mechanisms to overcome metal stress by enhanced production of osmolytes, efficient enzymatic and non-enzymatic antioxidant systems, sequestration of toxic metals into the vacuoles and effective compartmentalization^11–13^.

Several recent studies have demonstrated the role of phytohormones in combating environmental perturbations^7,14,15^. Jasmonic acid is a multifaceted hormone belonging to the family of plant growth regulators. It is produced as a consequence of lipoxygenases controlled process through oxygenation of unsaturated fatty acids^16^. Jasmonic acid is involved in a wide array of functions of plants mediating root growth, organ formation, storage, reproduction, fertility, ripening, senescence, signaling, etc.^17–19^. Under severe environmental constrains, JA is the core signaling molecule in several plant species like *Oryza sativa*, *Arabidopsis thaliana*, *Phaseolus lunatus and Solanum lycopersicum*^16,19^. Jasmonic acid stimulates plant immunity to combat the ill-effects of abiotic stresses on growth attributes and gene expression profiles within plants when applied externally in small quantities^20,21^.

A large family of tetracyclic diterpenoid compounds, gibberellins (GAs), is a group of plant growth hormones concomitant for plant growth, seed germination, water uptake, dormancy breaking and signaling^22,23^. Moreover, it has been reported by several workers that gibberellins promote plant growth under stress conditions. It is believed that gibberellins increase the synthesis of hydrolase in plants, relieve seed dormancy, improve seed vitality and seed germination, and are involved to repair damaged cell membranes^20,23,25^. A central role of gibberellins against different abiotic stresses is well-known in different studies^24,25^. Gibberellins act as potent signaling molecules to elevate growth and enhance developmental processes to counteract stress conditions and strengthen their immune system^26,27^. Additionally, little information is available regarding the role played by GAs in plants grown under stress conditions. Due to this reason, GAs are the prime targets of the present research.

Chickpea (*Cicer arietinum* L.) is one of the principal pulse crops. It is grown worldwide due to a rich source of proteins, minerals, vitamins, essential amino acids and carbohydrates^28^. It also comprises most vital components, well-known for their antioxidant activity to mediate reactive oxygen species (ROS) scavenging^29,30^. The production of chickpea is dwindling due to climatic changes for the last many years^31^. Cadmium stress has very well participated in decreasing the production of chickpea throughout the globe. Several recent studies demonstrated that leguminous crops are more susceptible to metal ion toxicity especially to Cd, in contrast to various plant species belonging to grasses and cereals. Therefore, leguminous crops including chickpea come across severe clampdown of biomass production even at lower metal concentrations^32^. However, little information is available with regard to tolerance and accumulation of metal ions in chickpea under Cd stress. Phytohormones have been the keen area of interest since recent past, however, the roles of GA_3_ and JA in Cd stress mitigation are not known well. Keeping in view the earlier mentioned plausible justification, the present study was undertaken to decipher the mechanism of Cd stress tolerance in chickpea under individual and/or combined application of JA and GA_3_.

## Materials and methods

### Plant growth and treatments

Authenticated chickpea seeds were taken from the local market and no specific permission is needed for their use as they are neither at risk of extinction nor endangered. In order to test the germination, the seeds were germinated in Petri plates (15 seeds/plate) lined with Whatman No 1 filter paper. This initial experiment showed more than 90% germination. The seeds were treated with 70% ethanol for 1 min for disinfection; thereafter the seeds were subjected to surface sterilization for 15 min with 4% sodium hypochlorite. The seed so treated was thoroughly rinsed for multiple times with double distilled water for avoiding any kind of chemical impurity. Seed priming of the sterilized seeds was carried out by placing the seeds in distilled water and then treating it with 1 nM JA for 12 h at 25 °C. Seed priming was executed by dissolving JA in absolute ethanol and then diluted to 1 nM concentration. For control, the seed priming was performed by placing the seeds in double distilled water for 12 h at 25 °C. Air-dried seeds (15 seeds/pot) were planted in earthen pots with dimension: (46 cm × 46 cm × 38 cm), each comprising 2 kg combined mixture of peat, perlite and sand in equal ratio (1:1:1). After germination, five seedlings were maintained in each pot. The experimental treatments were arranged in a randomized block design, with five replicates; each replicate consisted of 5 plants. The 11-day old seedlings were subjected to Cd stress (150 μM) by dissolving CdSO_4_·8H_2_O in nutrient solution. One M stock solution of CdSO_4_·8H_2_O was prepared and from this stock solution (150 µM) concentration was prepared by dissolving an appropriate amount in the nutrient solution consisting of 31 mg P L^−1^, 270 mg N L^−1^, 200 mg Ca L^−1^, 234 mg K L^−1^, 48 mg Mg L^−1^, 64 mg S L^−1^, 0.5 mg Mn L^−1^, 2.8 mg Fe L^−1^, 0.05 mg ZnL^−1^, 0.02 mg Cu L^−1^, 0.01 mg Mo L^−1^, 0.5 mg B L^−1^, and 0.1 mg Na_2_–Fe–ethylenediaminetetraacetic acid (EDTA) L^−1^. The control plants were supplied with the nutrient solution only. The Cd treatments (150) were applied every week till the end of the experiment (31 day). The stock solution of GA_3_ (10^–3^ M) was diluted to a concentration of GA_3_ (10^–6^ M) and applied in foliar form by mixing it in Tween-20 and supplied in alternate manner from the initial day of Cd treatment. The chickpea plants were raised under controlled conditions in a growth chamber with conditions such as 220 μmol m^−2^ s^−1^ photosynthetically active radiation, daytime/nighttime temperatures of 29/23 °C and relative humidity of 65–68%. The treatments were as follows:

- 0 Cd (Control)
- 0 Cd + JA (1 nM) as priming
- 0 Cd + GA_3_ (10^–6^ M) as foliar spray
- 0 Cd + JA (1 nM) + GA_3_ (10^–6^ M) as priming + foliar spray
- Cd (150 μM)
- Cd (150 μM) + JA (1 nM) as priming
- Cd (150 μM) + GA_3_ (10^–6^ M) as foliar spray
- Cd (150 μM) + JA (1 nM) + GA_3_ (10^–6^ M) as priming + foliar spray

### Plant growth, biomass and total chlorophyll estimation

Shoot and root lengths of the 35 day old chickpea plants were measured with a manual scale. In order to calculate dry weight (DW), the plant samples were completely desiccated at 70–75 °C for 48 h; the dried material was then weighed on a weighing balance. Chlorophyll measurements were carried out using the protocol of Arnon^33^. Fresh leaf samples (each 0.1 g), collected from five week old plants were homogenized in 10 mL of 80% acetone. The homogenate was centrifuged for 5 min at 10,000 rpm. The supernatant was collected in a clean tube and the process was repeated till the residue became colorless. The absorbance of the solution was read at 480, 645, and 663 nm against the acetone solution used as a blank.

### Estimation of cadmium

For the measurement of Cd, 0.5 g dried plant material was acid digested using HNO_3_ (nitric acid) and HClO_4_ (perchloric acid) in 4:1 ratio at 60 °C by adopting the hot plate block method depicted by Jackson^34^. The sample analysis for Cd was conducted through an atomic-absorption spectrophotometer (AAS) (Perkin Elmer AA700, USA). Cadmium translocation factor was determined as per the method of Bose and Bhattacharyya. Bioconcentration factor (BCF) was computed as heavy metal accumulated in each plant tissue to that dissolved in the soil medium as shown below:

1 $${\text{Shoot}}\;{\text{bioconcentration}}\;{\text{factor}}:\,{\text{BCF}}^{{\text{s}}} = {\text{C}}^{{{\text{shoot}}}} /{\text{C}}^{{{\text{soil}}}}$$

2 $${\text{Root}}\;{\text{bioconcentration}}\;{\text{factor}}:\;{\text{BCF}}^{{\text{r}}} = {\text{C}}^{{{\text{root}}}} /{\text{C}}^{{{\text{soil}}}}$$

Translocation factor (TF) of cadmium was computed using the above equations as follows:

$${\text{TF}} = {\text{BCF}}_{{{\text{shoot}}}} /{\text{BCF}}_{{{\text{root}}}}$$

### Determination of proline, glycine betaine and LRWC contents

Proline in fresh leaf samples was examined following the Bates, et al.^35^ method. For experimentation, a 0.5 g leaf sample was crushed with 10 mL 3% aqueous sulfosalicylic acid. The samples after filtering were measured for optical density at 520 nm using a spectrophotometer (Shimadzu UV-1800 UV, Kyoto Japan) and taking toluene as blank. Alongside, glycine betaine (GB) was also appraised according to the method of Grieve and Grattan^36^. The Leaf Relative Water Content (LRWC) was estimated using the following formula^37^:

$$LRWC=\frac{FW-DW}{TW-DW}\times 100$$

### Measurement of hydrogen peroxide (H_2_O_2_), lipid peroxidation (malondialdehyde) and electrolyte leakage

Hydrogen peroxide (H_2_O_2_) content in fresh leaf samples was determined as reported in Zhou, et al.^21^. Frozen leaves of both control and treated plants (each 0.25 g) were homogenized in an ice bath with 1 mL 0.1% (w:v) TCA. The homogenate was centrifuged at 12,000×*g* for 15 min at 4 °C. Aliquots of 100 μL from each tube were placed in 96-well plates and 50 μL of 10 mM potassium phosphate buffer (pH 7.0) and 100 μL of 1 M KI were added to each well. Commercial grade H_2_O_2_ was used to generate a standard curve. The plate was briefly vortexed, incubated at room temperature for 30 min. and the absorbance readings were taken at 390 nm in a micro-plate reader. The content of H_2_O_2_ was determined using the standard curve. Malondialdehyde (MDA) content (indicator of lipid peroxidation) was measured in the form of thiobarbituric acid reactive substances (TBARS) following the Heath and Packer^38^ protocol. Leaf samples (each 0.5 g) were homogenized in 10 mL 10% (w/v) trichloroacetic acid (TCA) solution on ice. The homogenate was centrifuged at 4000 rpm for 10 min at 4 °C, and then the supernatant was collected. Two mL of 0.6% thiobarbituric acid (TBA) were added to 2 mL aliquot of the supernatant. The mixture was kept in boiling water for 15 min and then rapidly cooled in an ice bath. After centrifugating at 4000 rpm for 10 min, the absorbance of the supernatant at 532 and 600 nm was measured. The concentration of MDA was calculated according to its extinction coefficient 155 mM^−1^ cm^−1^. Electrolyte leakage was determined as per Sutinen, et al.^39^ method. For the determination of electrolyte leakage, the upper fourth fully expanded leaves were excised from the control and treated plants and rinsed with distilled deionized water. Ten leaf discs (5 mm in diameter) were punched and subsequently placed in glass bottles each containing 15 mL of distilled deionized water. The bottles were then shaken at 300×*g* for 4 h in dark at 25 °C. After that, the electrolyte leakage (EC1) in the solution was measured with a conductivity detector (DDS SJ-308A, China) at 25 °C. The solutions were then boiled for 20 min and cooled to room temperature. The electrolyte leakage (EC2) of the boiled solution was measured at 25 °C. The below mentioned formula was used for calculating the electrolyte leakage (EL):

$$\text{Electrolyte leakage}\;\%= EC1/EC2\times 100$$

### Enzyme extraction and activity determination

For the preparation of enzyme extract and quantification of their activities, fresh plant material (each 1000 mg) was ground in a buffer solution prepared by mixing the following ingredients: 100 mM Tris-hydrochloride solution (pH-8), DTT (5 mM), 1.5 mM EDTA, MgCl_2_ (10 mM), PVP (1.5%), 5.0 mM magnesium acetate and aproptinin (1 μg mL^−1^). For the appraisal of activities of enzymes, the required amount of the supernatant was centrifuged for 15 min at 10,000×*g*. It should be noted that, for the extraction of APX, ascorbate (2 mM) was added separately to the buffer before its quantification.

Determination of superoxide dismutase (SOD; EC1.15.1.1) activity was carried out as per Ahanger, et al.^46^, based on the phenomenon of enzyme capability to hinder the light-dependent decrease in nitroblue tetrazoliumchloride (NBT). This sample mixture was mixed once, and the absorbance was taken at 560 nm. However, the amount of enzyme essential to undergo 50% inhibition for photo-reduction rate of NBT was considered to be one unit of SOD activity. The calculation was done as enzyme units (EU) per mg of protein.

Estimation of catalase (CAT; EC 1.11.1.6) activity was done as stated by Nakano and Asada^40^, by reducing H_2_O_2_ followed by measuring the alteration in absorbance of the reaction mixture at 240 nm for 3 min. The calculation was done as enzyme units (EU) per mg of protein.

Ascorbate peroxidase (APX; EC 1.11.1.11) activity was assessed by the protocol of Nakano and Asada^40^. The basis of measurement was reduction in the absorbance of the reaction mixture comprising H_2_O_2_ and ascorbic acid. Optical density of all treated samples was recorded at 290 nm for 2–3 min.

The glutathione reductase (GR; EC 1.6.4.2) activity was measured depending on the decline in absorbance of the reaction mixture comprising GSSG and NADPH at 340 nm for 3 min. This protocol was adopted from Foyer and Halliwell^41^. The enzyme activity was expressed as EU mg^−1^ of protein.

Determination of monodehydroascorbate reductase (MDHAR; EC1.6.5.4) activity was done following Hossain, et al.^42^. The modulation in the absorbance of the treated samples was recorded spectrophotometrically at 340 nm for 2–3 min. The activity of the enzyme was expressed as EU mg^−1^ protein.

The estimation of dehydroascorbate reductase (DHAR; EC 1.8.5.1) activity was done according to Hossain, et al.^42^. The OD of all samples was read at 265 nm for 2–3 min.

Glutathione-S-transferase (GST; EC 2.5.1.18) activity was measured following Hossain, et al.^42^. A change in absorbance at 340 nm was monitored for 1 min. The total protein contents in the plant enzyme extracts were estimated using the Coomassie brilliant blue G-250^43^.

### Non-enzymatic antioxidants

Freshly stored plant samples (each 0.5 g) were macerated each in 5 mL of 5% meta-phosphoric, centrifuged for 15 min at 11,500×*g* under refrigeration. The oxidized fraction was removed and reduced by adding 0.1 M dithiothreitol to determine the presence of total ascorbic acid (AsA). Total AsA and reduced AsA content were measured at 265 nm.

The estimation of dehydroascorbate (DHA) content was carried out by deducing the reduced AsA obtained from the total amount of AsA present. Estimation of reduced amounts of glutathione (GSH), glutathione disulfide (GSSG), and total glutathione (GSH + GSSG) was done following Griffith^44^. The determination of GSSG content was done after elimination of GSH by 2-vinylpyridine derivatization. The GSH quantification was done after deducting GSSG obtained from the total GSH content.

### Methylglyoxal (MG) content estimation

For estimating MG content, the procedure suggested by Wild, et al.^45^ was adopted. The *glyoxalase I* (Gly I; EC 4.4.1.5) activity of the samples was determined following Hossain, et al.^42^. Here, the extinction co-efficient of 3.37 mM^−1^ cm^−1^ was considered. *Glyoxalase II* (Gly II; EC 3.1.2.6) activity in the leaf tissues was carried out as stated by Hossain, et al.^42^. Here, the extinction co-efficient of 13.6 mM^−1^ cm ^−1^ was used.

### Estimation of nutrient elements

The dried samples of shoot and root were grounded to powder and then digested in sulphuric acid and nitric acid (1/5, v/v) for 24 h. After that the samples were treated with nitric acid and perchloric acid (5/1, v/v). The estimation of elemental content was performed by atomic absorption spectrophotometer (Analyst 300, Perkin-Elmer, Germany).

### Statistical analysis

The data of each variable was subjected to Statistix-10 for calculating one-way analysis of variance and Bonferroni test was employed to work out the differences among the mean values of all treatments at *P* ≤ 0.05. The values presented in tables or figures represent means ± standard error (SE).

## Results

### Growth, biomass yield

**Figure 1:**
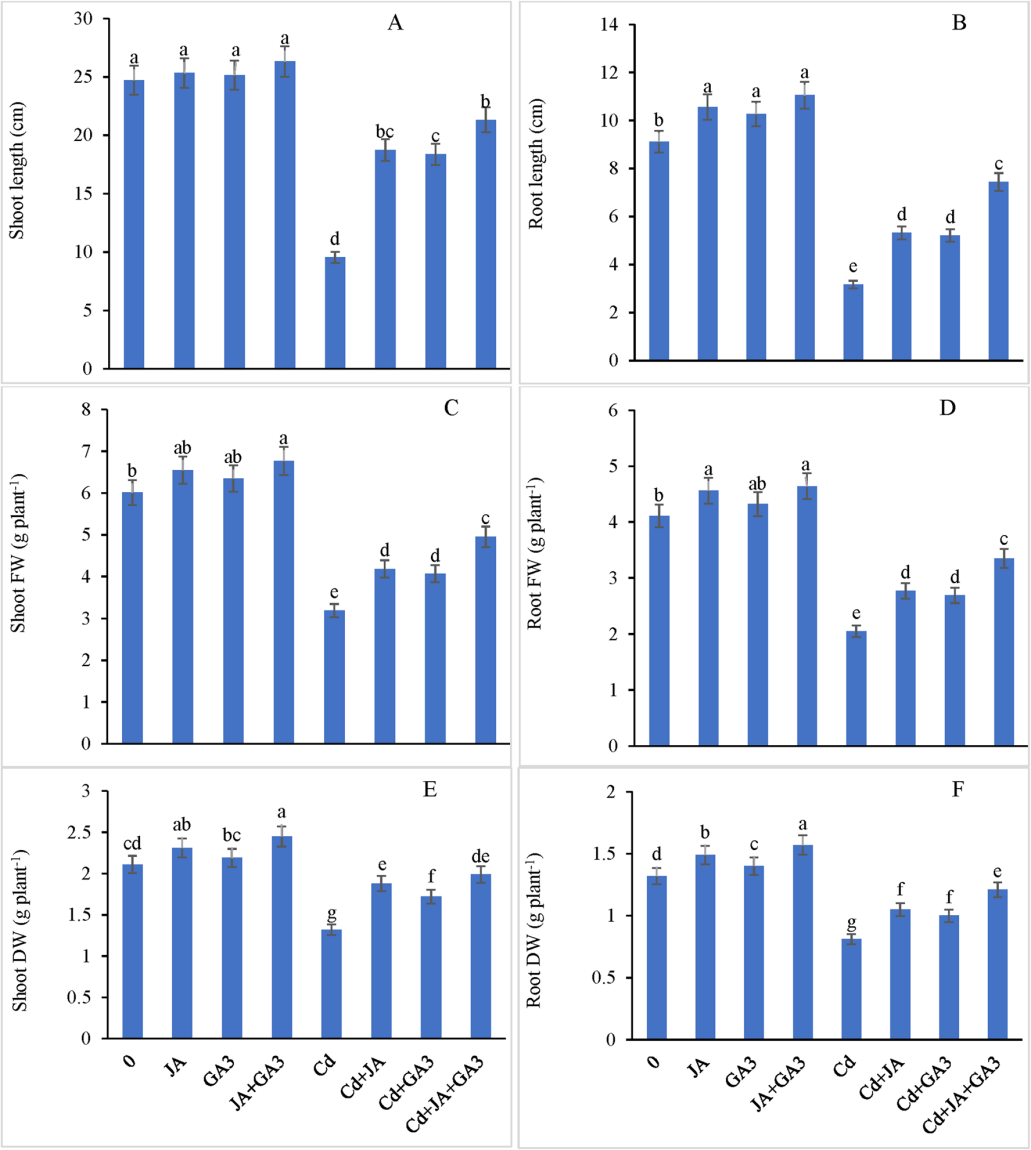
Ameliorating role of JA and GA_3_ applied individually and/or in combination on (**A**) shoot length, (**B**) root length (**C**) shoot FW, (**D**) root FW, (**E**) shoot DW and (**F**) root DW in Cd-stressed chickpea plants (Mean ± S.E.).

In the present study, Cd treatment reduced shoot height by 61.36% and root length by 65.24% with reference to the controls. However, JA treatment alone stimulated the shoot length by 54.45% and root length by 36.27%, whereas gibberellic acid ameliorated the Cd-induced injurious effects by inclining shoot length and root length by 50.36% and 32.80%, respectively, comparable to those in plants supplied with Cd only (Fig. 1A,B). Additionally, combined application of JA and GA_3_ to the Cd-treated chickpea plants improved the length of shoots by 70.89% and that of roots by 71.60%, respectively, with reference to plants treated with Cd only.

Cadmium stress decreased the shoot and root FW by 46.92% and 50.12%, respectively, over the corresponding controls. However, supplementation of JA or GA_3_ individually restored the FW, but the supplementation of the combined treatment of JA + GA_3_ to the Cd stressed plants enhanced the shoot FW by 55.17% and root FW by 63.41% comparable to those in the Cd-treated plants (Fig. 1C,D). A remarkable decrease of about 37.44% and 38.63% in shoot dry weight and root dry weight, respectively, was observed in the Cd-treated chickpea plants with respect to those in the controls. Individually applied JA to the Cd stressed plants elevated shoot as well as root dry weight by 42.42% and 29.62%, respectively. Moreover, individual GA_3_ application also enhanced shoot dry weight by 30.30% and root dry weight by 23.45% over those in the plants supplied with Cd only (Fig. 1E,F). The combined effect of JA + GA_3_ to the Cd exposed plants enhanced the shoot and root dry weights by 50.75% an 49.38%, respectively, over the controls, which is much higher than that shown by the individual effect of the either hormone. Although the PGRs applied individually had a beneficial effect in mitigating the injurious effects of Cd on various growth-related attributes in the chickpea plants, a significant interactive effect of the combined application of JA + GA_3_ + Cd was recorded for all growth variables (*P* ≤ 0.001), which indicates that both PGRs functioned synergistically to offset the adverse effects of Cd toxicity.

### Cd accumulation and translocation factor

Plants raised under Cd treatment showed Cd accumulation of 37.31 mg kg^−1^ DW within roots followed by 15.57 mg kg^−1^ DW in the shoots of the chickpea plants. Supplementing Cd stressed plants with JA decreased the Cd accumulation to 5.11 mg kg^−1^ DW in shoots and 15.72 mg kg^−1^ DW in roots (Table 1). However, the Cd stressed plants supplemented with GA_3_ also showed a decline in metal accumulation in the present study. The combinatorial supplementation of JA + GA_3_ to Cd supplemented plants lowered the Cd accumulation to 4.32 mg kg^−1^ DW in shoots and 14.31 mg kg^−1^ DW in roots, respectively. Translocation factor showed a declining trend under individual and combined application of JA and GA_3_. The TF was reduced from 0.417 to 0.325, and from 0.417 to 0.345 with JA and GA_3_, respectively. A further decrease from 0.417 to 0.301 was observed with the combined treatment of JA + GA_3_ to the Cd treated plants (Table 1). Overall, both PGRs supplemented singly, significantly reduced Cd accumulation in plant tissues, whereas a considerable synergistic effect of the joint supplementation of JA + GA_3_ + Cd was noticed for all Cd transport related attributes, which was evident from a significant interactive effect of these PGRs on the metal uptake and accumulation (*P* ≤ 0.001).

**Table 1:** Effect of JA and GA_3_ applied individually and/or in combination on Cd accumulation in shoot and root, and translocation factor in chickpea under Cd toxicity (Mean ± S.E.).

Treatments	Shoot Cd (mg kg^−1^ DW)	Root Cd (mg kg^−1^ DW)	Translocation factor (TF)
0	ND	ND	ND
JA	ND	ND	ND
GA_3_	ND	ND	ND
JA + GA_3_	ND	ND	ND
Cd	15.57 ± 0.63a	37.31 ± 1.41a	0.417 ± 0.01a
Cd + JA	5.11 ± 0.12b	15.72 ± 0.38b	0.325 ± 0.01bc
Cd + GA_3_	5.56 ± 0.14b	16.11 ± 0.40b	0.345 ± 0.01b
Cd + JA + GA_3_	4.32 ± 0.09b	14.31 ± 0.32b	0.301 ± 0.01c

### Pigment system

**Figure 2:**
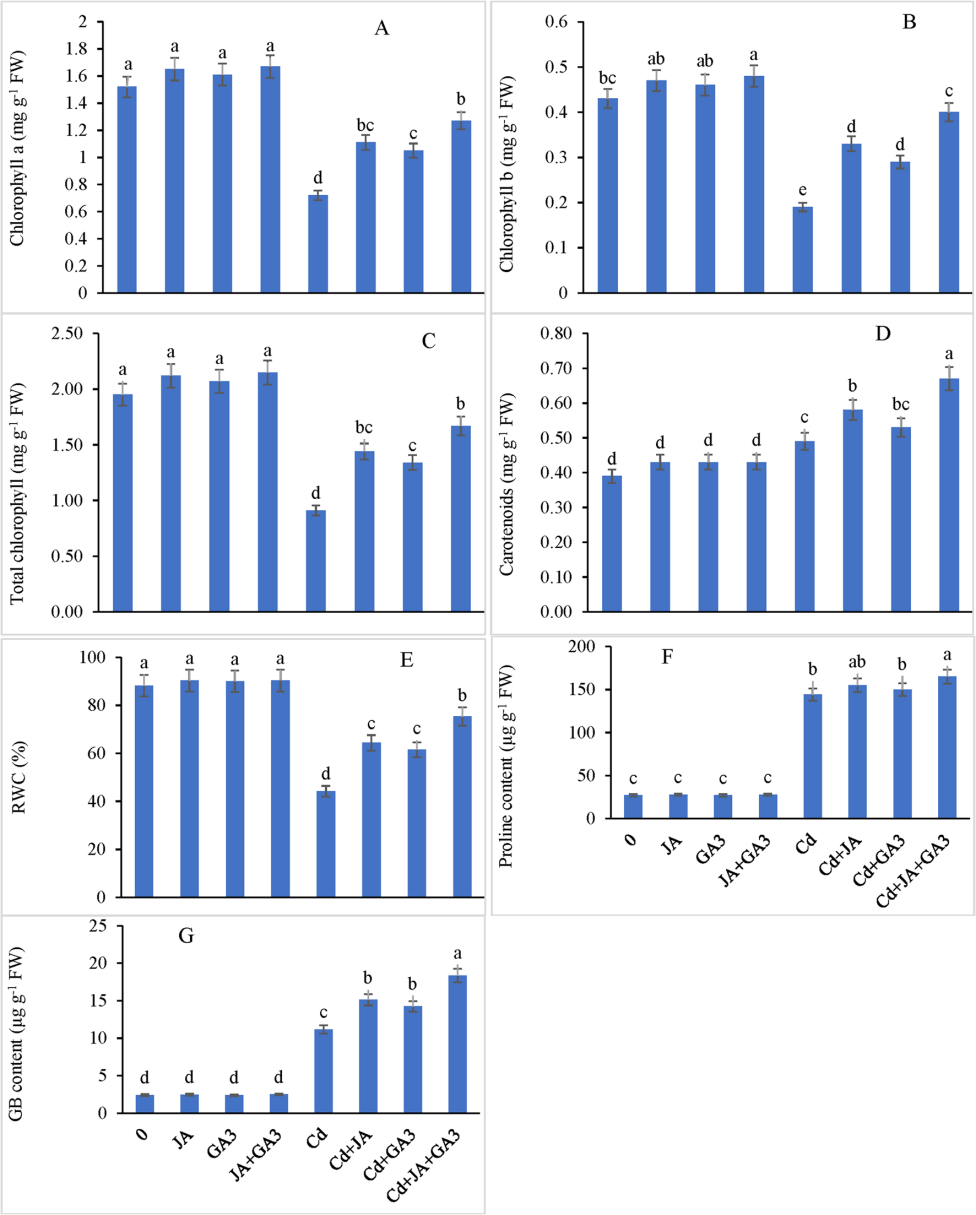
Restoration of (**A**) chlorophyll a, (**B**) chlorophyll b, (**C**) total chlorophyll, (**D**) carotenoid content, (**E**) RWC: relative water content, (**F**) proline content, and (**G**) GB: glycinebetaine in Cd-stressed chickpea plants by external supplementation of JA and GA_3_ applied individually and/or in combination (Mean ± S.E.).

The Cd application led to a significant reduction in Chl a, Chl b and total Chl by 52.63%, 55.81% and 53.33%, respectively, compared to the controls. Individual JA application enhanced Chl a by 54.16%, Chl b by 73.68% and total Chl by 58.24%, whereas GA_3_ supplementation also enhanced Chl a by 45.83%, Chl b by 52.63% and total Chl by 47.25% compared to those in the Cd-treated plants (Fig. 2A–C). Furthermore, the combined (JA and GA_3_) treatment had a prominent effect on pigment enrichment than that by either of the hormones applied individually. The carotenoid content increased by 25.64% with Cd stress compared to the controls, however, exogenously applied JA and GA_3_ to the Cd-stressed plants boosted the carotenoid synthesis by 18.36% and 8.16%, respectively, with respect to those in the plants stressed with Cd. The combined application of JA + GA_3_ further enhanced the carotenoid content by 36.73% in the Cd treated plants over the controls (Fig. 2D). Overall, individual or combined application of both PGRs showed a significant interactive effect (*P* ≤ 0.001) on reducing the injurious effects of Cd metal on different photosynthetic pigments.

### Relative water content, proline and GB content

In comparison with the controls, Cd stress decreased relative water content significantly by 49.84%, however, RWC was remarkably increased in the Cd-stressed plants supplied with JA and GA_3_. JA applied singly enhanced RWC by 45.55%, while GA_3_ ameliorated the toxic effects of Cd by increasing RWC up to 39.08% with respect to that in the controls (Fig. 2E). The combined supplementation of JA and GA_3_ was more effective in stress alleviation by increasing RWC content by 70.34% over the controls. With respect to the control, the Cd-stressed plants supplemented with JA and GA_3_ displayed no significant difference in proline and GB accumulation. The plants raised under Cd resulted in enhanced accretion of proline and GB by 5.31-fold and 4.65-fold correspondingly, compared with the controls (Fig. 2F,G). Proline and GB contents in the chickpea plants increased by 5.71-fold and 6.30-fold, respectively, under the individual application of JA to the Cd-stressed plants (Cd + JA). GA_3_ supplemented individually to the Cd-stressed plants also enhanced the proline content by 5.53-fold and GB by 5.92-fold over the controls. Additionally, a remarkable increase of 6.08-fold and 7.64-fold in proline and GB content, respectively, was recorded when the plants subjected to Cd were supplemented with JA and GA_3_ in combination (Cd + JA + GA_3_). Generally, a significant interactive impact (*P* ≤ 0.001) of both PGRs applied singly or jointly with Cd metal was recorded on all three physio-biochemical attributes.

### H_2_O_2_, MDA content and EL

**Figure 3:**
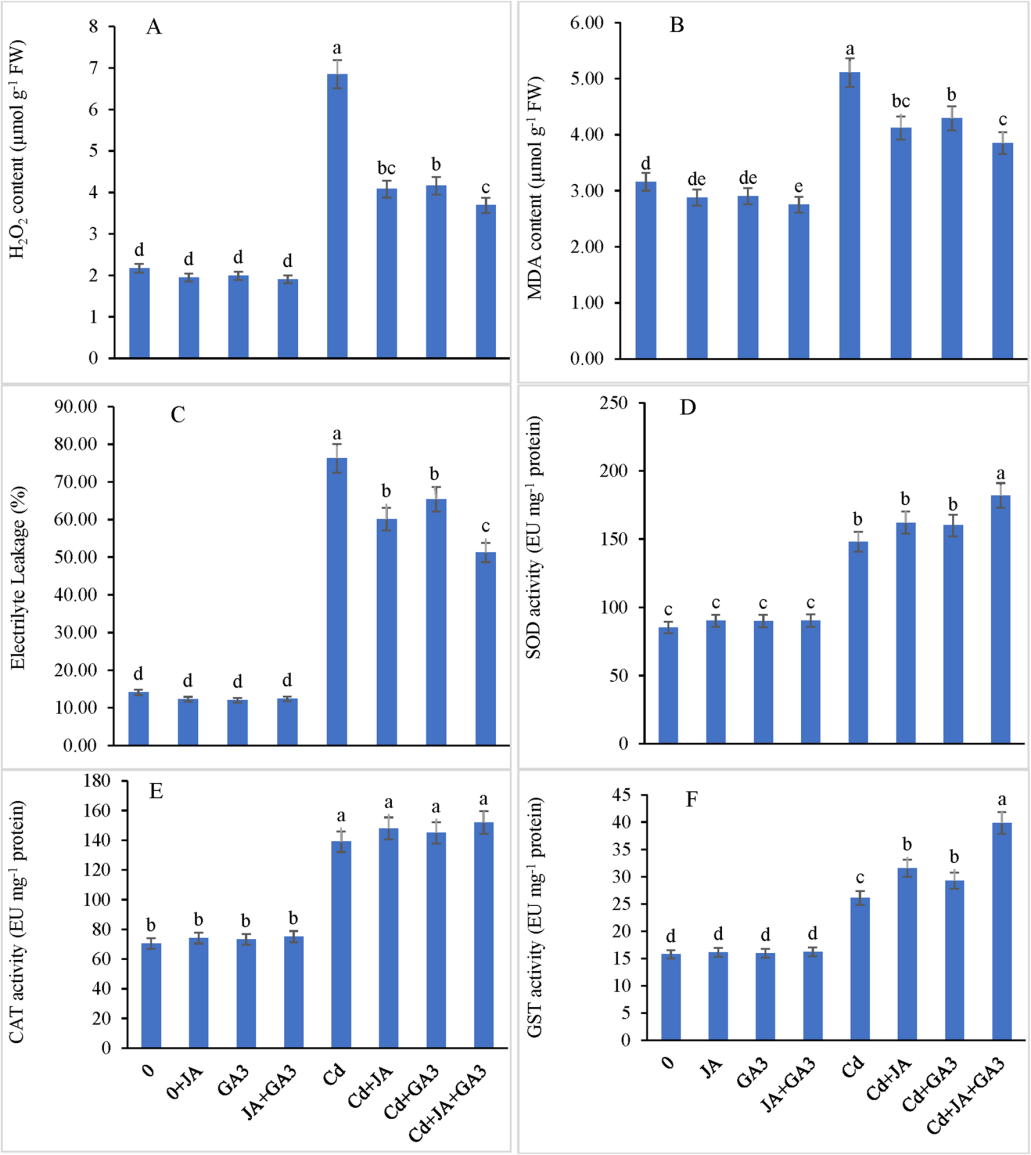
Effect of JA and GA_3_ applied individually or in combination decreased the oxidative stress biomarkers, (**A**) H_2_O_2_: hydrogen peroxide content, (**B**) MDA: malondialdehyde content, (**C**) EL: electrolyte leakage, (**D**) SOD: superoxide dismutase, (**E**) CAT: catalase and (**F**) GST: glutathione-*S*-transferase in Cd-stressed chickpea plants (Mean ± S.E.).

Cadmium stress increased H_2_O_2_, MDA and EL by 215.66%, 61.70% and 76.25% with reference to those in the controls. Plants exposed to Cd stress when supplemented with JA singly showed a decline in H_2_O_2_, MDA and EL by 88.01%, 30.37% and 60.11%, respectively, over the controls (Fig. 3A–C). Foliar application of GA_3_ decreased H_2_O_2_, MDA and EL by 91.70%, 35.75% and 65.35%, respectively, as compared to the controls. A sharp decline of 70.04%, 21.83% and 51.23% in H_2_O_2_, MDA and EL, respectively, was recorded in the Cd-treated plants after supplementation with the combined supply of JA and GA_3_ (Cd + JA + GA_3_), respectively. Both PGRs fed to the chickpea plants had a significant synergistic effect on reducing all three physio-biochemical attributes in the Cd-stressed chickpea plants, which was evident from a highly significant interaction of these PGRs with Cd (*P* ≤ 0.001).

### Antioxidant enzymes’ activities

Cadmium stress increased the SOD, CAT and GST activities by 73.76%, 97.47% and 65.54% when compared to those in the untreated plants. JA application to the Cd stressed plants showed a further increase in the activity of SOD (89.76%), CAT (110.25%) and GST (100.19%) compared with the controls. Individual application of GA_3_ to the Cd affected plants also caused an increase in the activity of SOD by 87.41%, CAT by 105.99% and GST by 85.72% compared with the controls (Fig. 3D–F). Conversely, the combined effect of JA + GA_3_ on the Cd-stressed plants further accelerated the activities of SOD, CAT and GST by 113.13%, 115.93% and 152.85%, respectively, relative to the controls.

#### Activities of the AsA-GSH pathway enzymes

**Figure 4:**
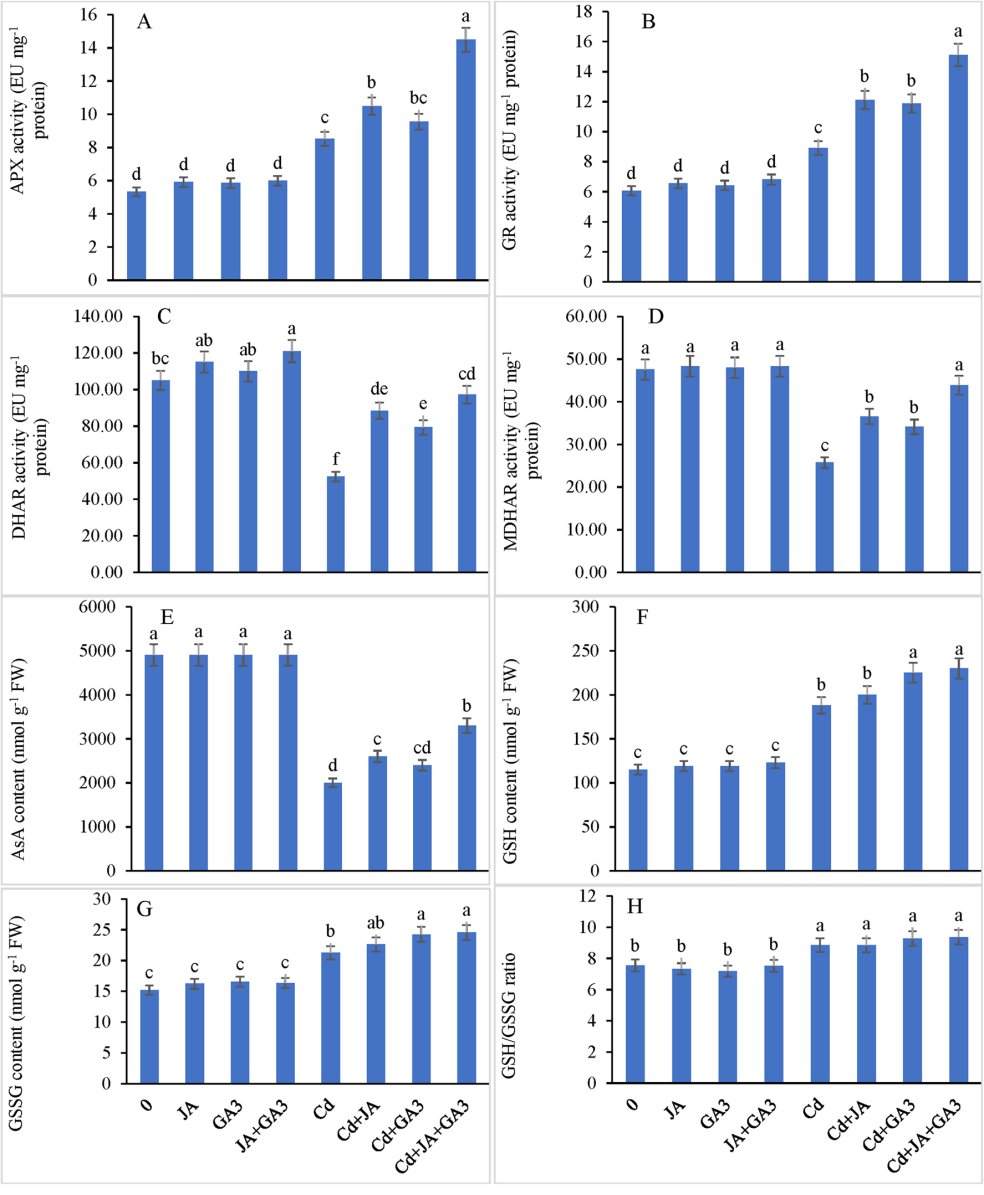
Supplementation of JA and GA_3_ individually and/or in combination enhanced the activities of the ascorbate–glutathione cycle enzymes, (**A**) APX: ascorbate peroxidase, (**B**) GR: glutathione reductase, (**C**) DHAR: dehydroascorbate reductase, (**D**) MDHAR: monodehydroascorbate reductase, (**E**) AsA: ascorbic acid content, (**F**) GSH: reduced glutathione, (**G**) GSSG: oxidized glutathione, and (H) GSH/GSSG ratio in Cd-stressed chickpea plants (Mean ± S.E.).

Under normal growing conditions, no significant difference in enzyme activities of the ASA-GSH pathway was observed in plants supplemented with JA and GA_3_. As compared to the control, the plants experiencing Cd stress showed higher APX activity (59.66%), and the activity was enhanced by 96.81% and 79.17% on individual supplementation of JA and GA_3_, respectively, to the Cd-stressed plants (Fig. 4A). Higher APX activity (171.85%) was observed in Cd-treated chickpea plants when JA and GA_3_ were applied in combination. While comparing with the controls, the GR activity was found to be triggered by 46.78% in the Cd-stressed chickpea plants (Fig. 4B). Furthermore, JA-pre-treated Cd stressed plants (Cd + 1 nM JA) showed increased GR activity by 99.50%, whereas GA_3 _+ Cd increased the GR activity by 95.71% compared with the controls. The combined application of JA and GA_3_ further enhanced the GR activity by 148.92% in the Cd treated plants with respect to the control. Cadmium stress decreased the activity of DHAR by 46.78% and MDHAR by 45.90% compared with the controls (Fig. 4C,D). However, JA-priming resulted in a less decrease in DHAR and MDHAR activities by 15.08% and 23.11%, respectively, under Cd stress. Foliar addition of GA_3_ in Cd-supplemented plants also recorded less decrease in the activity of DAHR by 24.45% and MDHAR by 28.26%. The combined application of JA and GA_3_ to the Cd-stressed plants showed a minimal decrease by 7.36% and 7.69% in DHAR and MDHAR activity, respectively, compared to the respective controls. From the analysis of variance of the data for all the afore-mentioned enzymes, it is evident that both PGRs functioned synergistically (Interaction term JA + GA_3_ + Cd, *P* ≤ 0.001) to modulate the activities of these enzymes under Cd toxicity.

#### JA maintained the contents of ascorbic Acid (AsA), GSH, and GSSG, and GSH/GSSG ratio

During normal growth conditions, no significant effect of JA and GA_3_ was recorded on AsA levels. The AsA levels showed a sharp decline by 59.18% relative to the control when the plants were subjected to cadmium stress (Fig. 4E). Furthermore, the AsA levels further increased in the cadmium treated plants when supplemented with JA and GA_3_ either alone or in combination. However, the combined treatment of JA + GA_3_ showed a maximal enhancement in AsA levels by 65% in the plants subjected to Cd stress relative to those of Cd-alone treated plants. An increase of 63.47% was observed in GSH concentration in the plants under Cd stress, relative to the control (Fig. 4F). Jasmonic acid pre-treatment and foliar supplementation of GA_3_ to the Cd-stressed pants showed a further escalation in GSH content by 6.38% and 19.68%, respectively, compared with the controls. Further, the Cd affected plants supplemented with JA + GA_3_ in combination showed enhanced GSH activity by 22.34% with regard to that in Cd-alone treated plants.

Cadmium metal toxicity enhanced the GSSG content by 39.57% relative to the control (Fig. 4G). However, further enhancements by 6.50% and 14.08% were observed by the individual supplementation of JA and GA_3_ to the Cd treated plants compared with the controls. The combined treatment of JA + GA_3_ to Cd-supplemented plants exhibited a further enhancement in the GSSG content by 15.54% with regard to the control. The GSH/GSSG ratio increased by 17.06% under Cd stress. However, exogenously applied JA and GA_3_ individually as well as in combination did not show any significant effect on the GSH/GSSG ratio (Fig. 4H). All the afore-mentioned variables were synergistically regulated by the combined supplementation of both PGRs (*P* ≤ 0.001), indicating that the negative impact of the metal was alleviated markedly on these attributes.

#### Methylglyoxal (MG) and glyoxalase system

**Figure 5:**
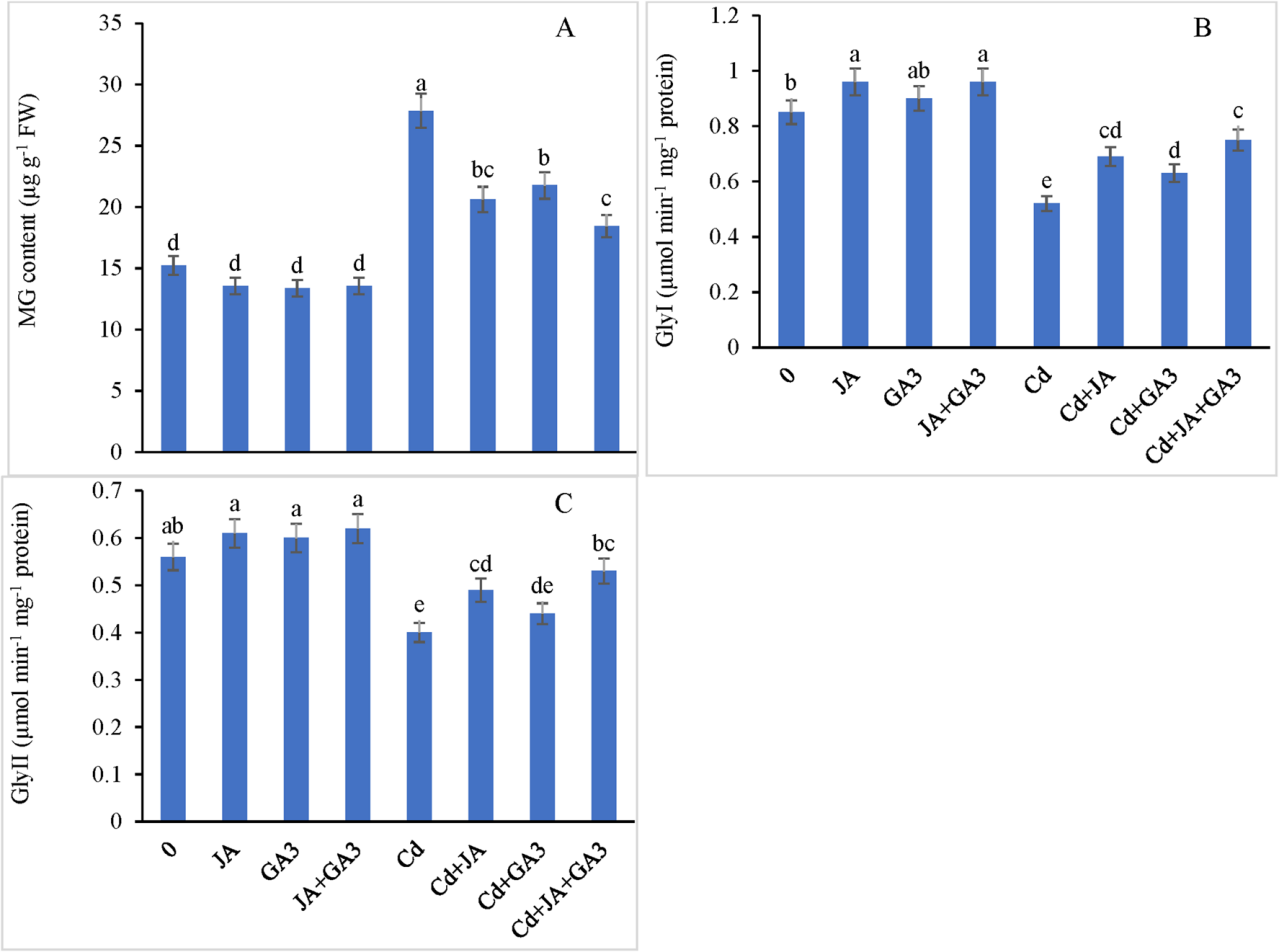
Supplementation of JA and GA_3_ individually and/or in combination decreased the (**A**) methyglyoxal (MG) content, and boosted the activities of the glyoxalase system enzymes (**B**) GlyI and (**C**) GlyII in Cd-stressed chickpea plants (Mean ± S.E.).

Higher levels of MG (82.98%) were observed in the plants stressed with Cd compared with the control. However, the Cd-stressed chickpea plants treated with JA and GA_3_ individually showed a significant reduction by 25.96% and 21.86% compared with the controls (Fig. 5A). The cadmium stressed plants illustrated a maximal decrease (33.78%) in the MG level when supplemented with the combined application of JA + GA_3_ with respect to the controls. The GlyI and GlyII activities declined by 38.82% and 28.57%, respectively, under Cd stress over the controls (Fig. 5B,C). Relative to those in the Cd-treated plants, JA-pretreatment suppressed the activities of both GlyI (32.69%) and GlyII (22.50%) enzymes in the Cd stressed plants. Foliar application with GA_3_ to the Cd-stressed plants also caused an improvement in the GlyI and GlyII activities by 21.15% and 10%, respectively, over the controls. Higher GlyI activity by 44.23% and that of GlyII by 32.50% were observed when JA + GA_3_ was applied in combination to the Cd stressed plants over the controls.

#### Mineral elements

**Figure 6:**
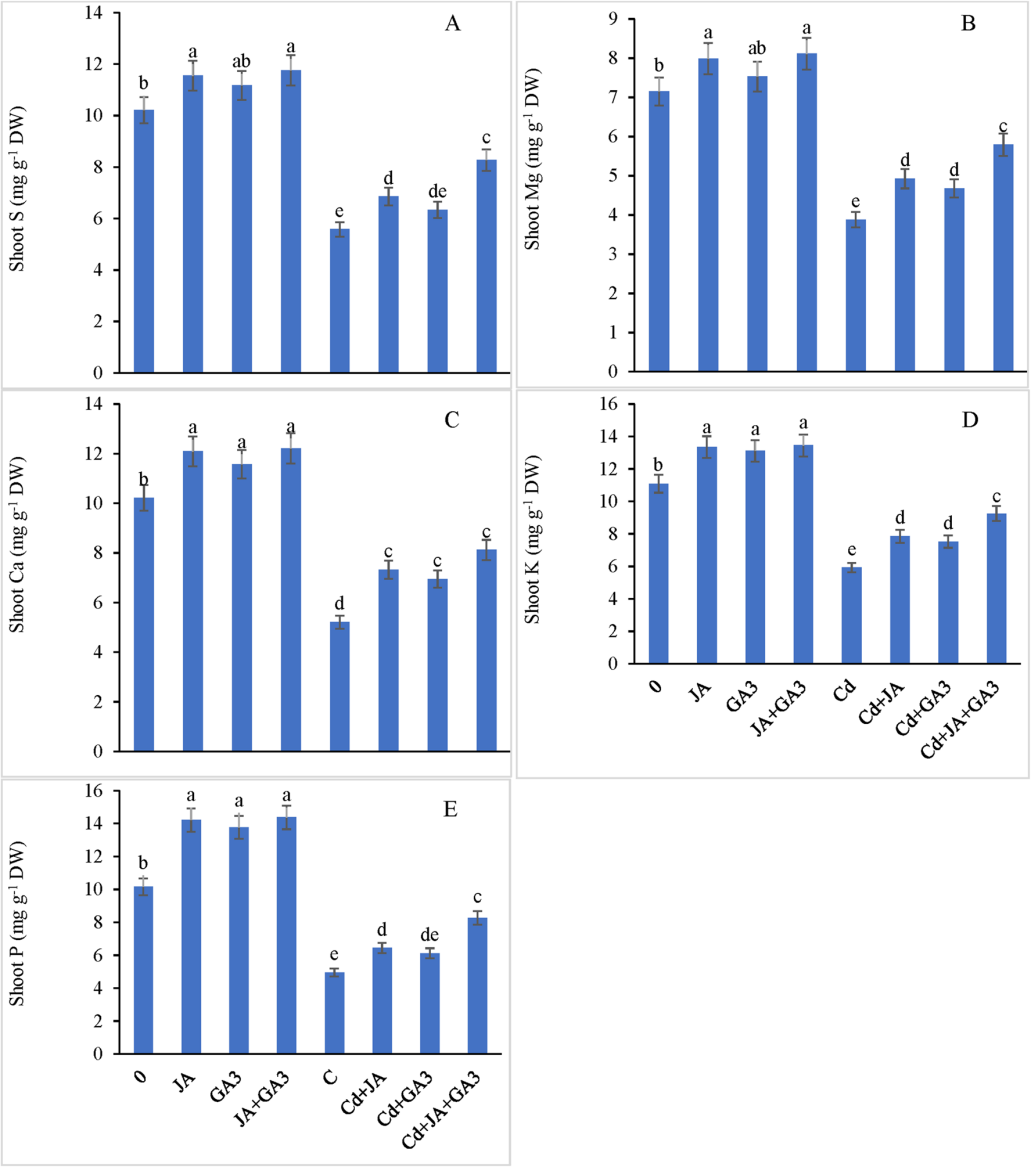
External supplementation of JA and GA_3_ individually and/or in combination restored the shoot mineral elements; (**A**) sulfur (S), (**B**) magnesium (Mg), (**C**) calcium (Ca), (**D**) potassium (K), and (**E**) phosphorus (P) in Cd-stressed chickpea plants (Mean ± S.E.).

**Figure 7:**
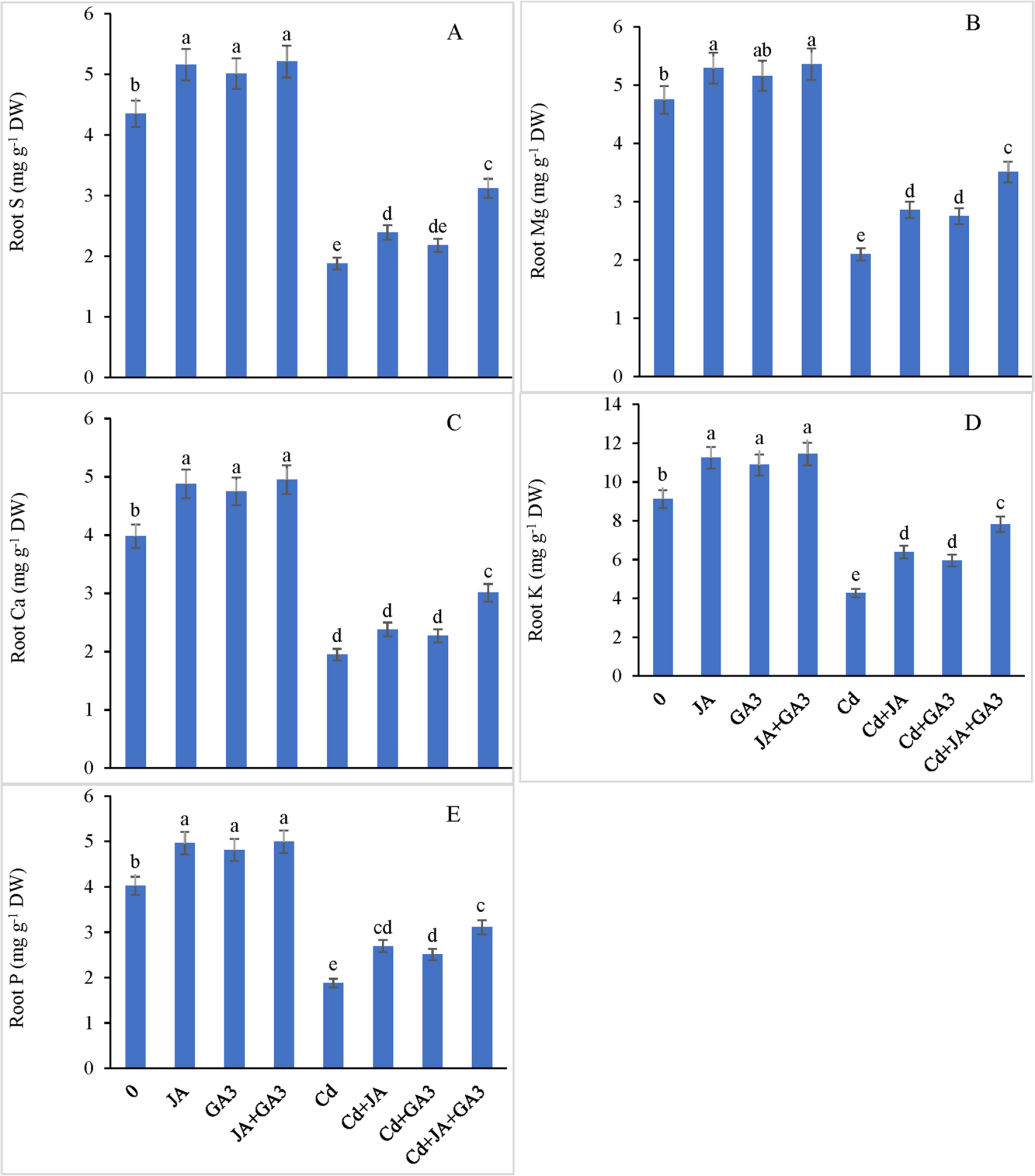
External supplementation of JA and GA_3_ individually and/or in combination restored the root mineral elements, (**A**) sulfur (S), (**B**) magnesium (Mg), (**C**) calcium (Ca), (**D**) potassium (K), and (**E**) phosphorus (P) in Cd-stressed chickpea plants (Mean ± S.E.).

As evident from the results, the shoot mineral accumulation declined under Cd stress. The shoot S, Mg, Ca, K and P contents were declined by 45.34%, 45.73%, 49.02%, 46.61% and 51.23%, respectively, with regard to the respective controls (Fig. 6A–E). JA priming to Cd-stressed plants increased the accumulation of S by 22.75%, Mg by 26.80%, Ca by 40.49%, K by 32.60%, and P by 30.10% compared with the controls. Cadmium stressed plants supplemented with GA_3_ increased the shoot S, Mg, Ca, K and P contents by 13.62%, 20.61%, 33.39%, 27.02% and 23.63%, respectively, compared with the controls. The combined application of JA + GA_3_ to the Cd-treated plants further enhanced the contents of shoot S by 48.20%, Mg by 49.22%, Ca by 55.85%, K by 56.25%, and P by 67.07% with respect to the controls. Similarly, root S, Mg, Ca, K and P contents were found to be decreased significantly under Cd stress by 56.78%, 55.78%, 51.00%, 53.17% and 53.23%, respectively, compared to the controls (Fig. 7A–E). JA and GA_3_ applied singly increased root S, Mg, Ca, K and P contents by 27.12%, 36.19%, 22.05%, 49.64%, 43.08% and 15.95%, 41.02%, 16.41%, 39.34%, 33.51%, respectively, in the chickpea plants under Cd stress with respect to the controls. However, the combined application of JA + GA_3_ further enhanced the accumulation of root S by 65.95%, Mg by 67.14%, Ca by 54.35%, K by 83.13%, and P by 65.42% in the Cd-stressed plants. Although the PGRs supplied singly had a positive impact to assuage the deleterious effects of Cd on all mineral nutrients determined in the chickpea plants, a significant interactive effect of the combined application of JA + GA_3_ + Cd was found for all mineral nutrients (*P* ≤ 0.001), which shows that both PGRs worked synergistically to improve the uptake and accumulation of all beneficial inorganic nutrients under Cd toxic regimes.

## Discussion

Due to its extremely toxic nature, Cd has widely been reported to impede normal metabolic processes and functioning of plants^46–48^. During the present study, chickpea plants exposed to Cd adversity displayed reduced growth and biomass production. The primary reasons for Cd-induced decline in growth have been reported in different studies, e.g., reduced photosynthetic machinery^8,24^, and restricted water and mineral uptake by roots^48,49^. However, improved growth and biomass in chickpea were observed upon seed priming with JA and foliar application with GA_3_ alone or in combination, indicating their synergistic role in promoting growth and biomass yield under stress conditions. Analogously, foliar-applied GA_3_ increased growth and biomass yield in lettuce by promoting cell division and elongation^24,50^. GA_3_-induced growth promotion can be ascribed to increased biomass by promoting synthesis of DNA, RNA, nuclear material and proteins^51,52^, improved enzyme activity and optimal nutrient uptake^2,24^ Combined application of JA and GA_3_ displayed more pronounced effect on growth and biomass yield in the current study that could be related to enhanced enzyme activities, higher accumulation of osmoprotectants and low metal translocation under cadmium stress.

Being highly mobile, Cd in plants gets accumulated in shoot and root^53^. During our study, we found higher Cd accumulation in roots than in shoot. It is believed that metal toxicity in plants directly depends on the amount of metal accumulating in plant parts, and its distribution and partitioning in different organs^14,53^. Notably, roots stand as the principal site of plants to come in direct contact with metal ions, which in turn regulate their flux inside the root tissues^54,55^. Nonetheless, chickpea roots accrued maximal quantities of Cd under Cd toxic regimes, under both the conditions of supply or non-supply of JA and GA_3_. However, the Cd-stressed plants treated with JA and GA_3_ accumulated very low quantity of Cd in both roots and shoots, so as to minimize Cd-induced impairments thereby boosting the overall growth of the chickpea plants. Possibly, JA mediated the biosynthesis of various organic acids in the root exudates or they might have also released thiol-like compounds for effective sequestration of Cd in the roots as a defensive mechanism^56^. Likewise, a similar kind of trend was also observed in the studies involving Cd and Cu metal ions^14^.

### Photosynthetic pigments

The extent of damage to the photosynthetic system from various environmental perturbations can best be explained through the disruption of photosynthetic pigments^8,24^. In response to Cd stress, severe reductions in chlorophyll and carotenoid pigments were observed during the present study. Reduction in chlorophyll and carotenoid pigments might have been due to the destruction of enzymatic machinery of the chlorophyll biosynthesis pathway and photosystem damage^57,58^, down-regulation of enzymatic activity^59^, and replacement of Mg^2+^ with Cd^2+^ in chlorophyll structures^60^. Usually, JA shields chlorophyll pigments under adverse metal conditions^14,58^. For example, JA application increased chlorophyll synthesis in *Capsicum frutescens* raised on Cd amended soils^61^, *Glycine max* under Ni stress^62^, and alfalfa under Cu Stress^14^. JA-mediated enhancement in photosynthetic pigments in the present study could be attributed to the synthesis of numerous secondary metabolites such as alkaloids, phenolics, and anthocyanins in various plant species^56,58^, which in turn can reduce Cd uptake and promote root mitotic activity^62^. Moreover, the application of GA_3_ stimulated chlorophyll synthesis in the chickpea plants raised on high Cd regimes. Our results corroborate well with those of Falkowska, et al.^63^ in *Chlorella vulgaris,* and Hasan, et al.^24^ in *Vigna radiata.* An increased pigment content under Cd stress upon foliar application of GA_3_ might have been due to limited oxidative damage and proper growth with enhanced antioxidant mechanisms.

### Relative water content, and proline and GB contents

RWC is considered to be one of the prospective stress indicators^64,65^. Exposure of chickpea plants to Cd resulted in decreased RWC in the present investigation. Several workers have shown significant changes in RWC under Cd ion toxicity in different plant species such as *Vigna aconitifolia* and *Phaseolus vulgaris*^66,67^ which were ascribed to high assimilation rate of metal ions in roots, so as to safeguard the upper plant parts from high accumulation of Cd by promoting optimal water uptake to the aerial parts^56,64,68^. Jasmonic acid counteracted the negative effects of Cd by increasing RWC in the chickpea plants in the present investigation. For example, supplementation of JA improved RWC content in *Phaseolus coccineus* and wheat plants^69,70^. Similarly, foliar applied GA_3_ alleviated the severe effects of Cd by augmenting RWC levels through reduction in metal accumulation^24,71^, increased root elongation and shoot biomass, and enhanced enzyme activities^72^.

Plants under stressful environments accumulate several compatible molecules like GB, proline and sugars to overcome osmotic stress triggered by several abiotic stress conditions^73,74^. Excessive proline amassing under stress helps plants to maintain redox potential and help in osmotic adjustments for stabilizing cellular structures and ROS scavenging^75,76^. Increased proline levels were observed in the chickpea plants upon priming with JA similar to that of Conrad, et al.^77^ who reported up-regulation of the proline rich proteins in the cells, thereby protecting the cells from ferroptosis by quenching free radicals. GA_3_ also improved proline levels that overcame the adversity created by salinity via regulating membrane permeability and improving macro- as well as micro-nutrients^24,78^. GB is widely known to maintain cellular osmo-regulatory processes in various plant species under different stress conditions^79–81^. GB is also believed to be involved in ROS quenching, safeguarding the photosynthetic machinery, up-regulating the stress-responsive genes, and preserving protein conformations^79,80^. Moreover, the application of both hormones promoted the accumulation of GB in the Cd-treated chickpea plants. Likewise, externally supplied JA was reported to considerably trigger betaine levels in *Glycine max* plants under Ni stress^62^, and *Vicia faba* and *Mentha arvensis* under Cd stress^82,83^. It could have been mainly due to up-regulation of BADH (betaine aldehyde dehydrogenase) in plants under metal stresses^79,84^.

### Cadmium stress enhanced H_2_O_2_ production, MDA levels and electrolyte leakage

Cadmium stress imposed to the chickpea plants induced enhanced H_2_O_2_ production, although under stressful conditions H_2_O_2_ is known to play a pivotal role in signaling^57,85^; conversely, it is also deliberated to be the chief ROS molecule negatively affecting plant cells^11^. Cd stress causes lipid peroxidation and enhances MDA production, which in turn perturbs the fluidity and integrity of biological membranes^86,87^, besides these, H_2_O_2_ also obstructs the Calvin cycle thereby causing perturbance in the rates of photosynthesis^85^. However, application of both JA and GA_3_ reduced the levels of both H_2_O_2_ and MDA in the Cd-stressed chickpea plants in the current investigation. JA is known to shield the cell membrane components by recovering the plants from lipid peroxidation induced by Cd stress^82,88^. JA is known to cause reduced accumulation of H_2_O_2_ and MDA by increasing antioxidant activities that help quench the ROS^20,89^. Our results are in agreement with those of Hasan, et al.^24^, and Hanaka, et al.^70^ who demonstrated that JA and GA_3_ application improved RWC, and decreased EL, and production of H_2_O_2_ and MDA under Cd stress.

### JA and GA_3_ improved antioxidant activity under Cd stress

Oxidative burst-mediated deterioration of plant tissues is mainly initiated by the ROS in plants. ROS production and antioxidative enzymes imbalance in cells during metal toxicity in plants, inevitably resulting in oxidative stress that leads to several physiological disorders^3,8^. Several studies regarding ROS mediated deterioration of proteins, nucleic acids and lipids have been carried out recently^1,90^. To counteract such situations, a robust antioxidant system based on enzymatic and non-enzymatic antioxidants exists in plants for redox balance^91,92^. Analogous to our study, several recent studies have demonstrated reduced modulations of antioxidant enzymes under Cd stress conditions^24,90,93^*.* However, JA and GA_3_ application modulated the AsA-GSH enzyme cycle differently, through altering the activities of APX, MDHAR, and DHAR to a varying level. Moreover, JA and GA_3_ treatment caused an enhancement in GR activity. It can mediate a well sustained redox status probably by renewing GSH from GSSG, which is well supported with the upsurge in GSH levels along with GSG/GSSG ratio. It has also been demonstrated that plants thriving under different environmental perturbations may activate the GSH dependent defense mechanisms that play a pivotal role in plant protection^91,94^. The AsA-GSH associated defense mechanism under JA and GA_3_ supplementation demonstrated further increased levels of GSH. This might have been the reason behind the biosynthesis and modulation of GR activity under Cd stress; this might have subsequently contributed in GST and GPX-facilitated effectual decontamination of hydroperoxides. Our results are in agreement with those of Gangwar, et al.^95^ who demonstrated an increase in the AsA-GSH cycle enzymes in *Pisum sativum* under chromium stress. Similar findings were also reported by Hassan and Mansoor^96^ in mung bean. Some other authors have also demonstrated an upsurge in the AsA-GSH pathway enzymes under different environmental stresses^97,98^.

### JA and GA_3_ modulated the glyoxylase cycle to alleviate Cd toxicity

Due to pertinent involvement in redox balance and MG detoxification, the GSH-dependent Gly system was studied in the present study to decipher its role in improving Cd stress tolerance under the effect of JA and GA_3_ application. The study revealed that the MG concentration under Cd stress was enhanced possibly because of the non-significant activities of the glyoxylase enzymatic system (both Gly I and Gly II enzymes). Furthermore, impaired activity of Gly II may also enhance GSH in a way that the resultant *S*-lactoylglutathione accumulation may become extremely cytotoxic^42^. JA and GA_3_ application reversed the toxic effects of MG by enhancing the activities of GlyI and GlyII enzymes, which in turn might have protected the plant cells from the cytotoxic effects of MG^99^. Our findings are in line with those of Hossain, et al.^42^ in mung bean. Increased Cd stress tolerance in the chickpea plants under JA and GA_3_ application can be attributed to higher antioxidant enzyme activities (APX, GPX, and GST), improved Gly enzyme (Gly I and II) activities, increased nutrient uptake, and efficient ROS detoxification.

### JA and GA_3_ reduced metal uptake and increased nutrient accumulation under Cd stress

The present study demonstrated a reduced mineral uptake which corroborates with other studies^46,82,100^. The reduced uptake of minerals may be attributed to the competition of Cd with other toxic metals for the same transporters^101^. Addition of JA to plant growth medium can reduce the Cd buildup and boost mineral uptake of ions like, Ca, Na, Mg, K, Cu, P and Fe^102^. JA and GA_3_ applied either independently or in combinatorial form reduced the Cd translocation and increased the nutrient uptake by the plants possibly through the restoration of H^+^-ATPase activity^102^, maintenance of osmotic potential^103^, and enhancement in antioxidant machinery^103,104^. Additionally, application of GA_3_ was reported to also improve growth, photosynthetic activities, and mineral nutrient uptake in *Glycine max*^105^. Taken together, our results show that application of JA and GA_3_ alleviated the Cd toxicity in the chickpea plants by reducing Cd uptake, improving pigment content, enhancing osmolytes’ accumulation, and modulating the key enzymes of the ascorbate glutathione pathway and glyoxylase cycle.

## Conclusions

The present study elucidated that the Cd toxicity in the chickpea plants led to decreased biomass, pigment concentrations, and RWC, but it enhanced MDA levels and triggered ROS production. However, supplementation of JA along with GA_3_ proved to be a viable approach for enabling the chickpea plants to thrive well under Cd stress. The supplementation of JA and GA_3_ improved photosynthetic characteristics through pigment protection, and maintenance of water balance. Apart from these functions, the combined application of JA and GA_3_ proved to be very effective in enhancing osmolyte accumulation and effectively detoxifying the ROS through improving the capabilities of both enzymatic as well as non-enzymatic antioxidants to avert oxidative damage. Moreover, the combined JA and GA_3_ application played a pivotal role in maintaining the AsA/DHA and GSH/GSSG ratios that also helped to reduce the oxidative damage through regulation of the GSH-based Gly systems to detoxify MG. Additionally, seed priming with JA and foliar application of GA_3_ reduced the Cd accumulation and enhanced the uptake of essential mineral nutrients in the chickpea plants. The synthetic plant growth regulators tested in the current study are quite costly, so before recommending to the farmers for their widespread use under natural field conditions, there is a need to work out benefit/cost ratio of each PGR. Nonetheless, the concentrations used of both JA and GA_3_ in the present study for seed priming and foliage spray are very low, so very little amount of both PGRs have been used that have shown alarming results in terms of counteracting the adverse effects of Cd on chickpea plants. Moreover, since seed priming technique does not require much amount of a PGR, so this technique, if effective in offsetting the stress-induced adversity, can be of widespread use by the farmers.

## Supplementary Information

Supplementary Information.
